# Supplementary material for: Role and mechanism of NCAPD3 in promoting malignant behaviors in gastric cancer
Source: Front Pharmacol. 2024 Apr 22;15:1341039. doi: 10.3389/fphar.2024.1341039 (PMC11070777; doi:10.3389/fphar.2024.1341039)
Supplement: Supplementary file 11 [file DataSheet2.ZIP › GSEA/Transcipt factor/my_analysis.Gsea.1599461814734/FOXO1_02.html]

Details for gene set FOXO1\_02[GSEA]

|  || Dataset | filtered\_dataset.sample\_info.cls#WT\_versus\_NCAPD3\_MUT |
| Phenotype | sample\_info.cls#WT\_versus\_NCAPD3\_MUT |
| Upregulated in class | NCAPD3\_MUT |
| GeneSet | FOXO1\_02 |
| Enrichment Score (ES) | -0.4715612 |
| Normalized Enrichment Score (NES) | -2.374564 |
| Nominal p-value | 0.0 |
| FDR q-value | 0.011868928 |
| FWER p-Value | 0.044 |
Table: GSEA Results Summary

  

Fig 1: Enrichment plot: FOXO1\_02      
 Profile of the Running ES Score & Positions of GeneSet Members on the Rank Ordered List

  

| SYMBOL | TITLE | RANK IN GENE LIST | RANK METRIC SCORE | RUNNING ES | CORE ENRICHMENT || 1 | 23338 | JADE2 | 71 | 0.855 | -0.0008 | No |
| 2 | 221504 | ZBTB9 | 237 | 0.647 | -0.0819 | No |
| 3 | 6426 | SRSF1 | 243 | 0.637 | -0.0479 | No |
| 4 | 1979 | EIF4EBP2 | 532 | 0.456 | -0.2291 | No |
| 5 | 4784 | NFIX | 567 | 0.439 | -0.2278 | No |
| 6 | 84335 | AKT1S1 | 572 | 0.437 | -0.2049 | No |
| 7 | 26065 | LSM14A | 636 | 0.405 | -0.2264 | No |
| 8 | 7067 | THRA | 976 | -0.392 | -0.4484 | Yes |
| 9 | 9572 | NR1D1 | 977 | -0.392 | -0.4252 | Yes |
| 10 | 51667 | NUB1 | 978 | -0.392 | -0.4020 | Yes |
| 11 | 687 | KLF9 | 996 | -0.402 | -0.3905 | Yes |
| 12 | 10938 | EHD1 | 1019 | -0.419 | -0.3817 | Yes |
| 13 | 23764 | MAFF | 1039 | -0.436 | -0.3697 | Yes |
| 14 | 3918 | LAMC2 | 1088 | -0.466 | -0.3768 | Yes |
| 15 | 54541 | DDIT4 | 1133 | -0.502 | -0.3789 | Yes |
| 16 | 639 | PRDM1 | 1159 | -0.523 | -0.3661 | Yes |
| 17 | 2119 | ETV5 | 1176 | -0.545 | -0.3454 | Yes |
| 18 | 153222 | CREBRF | 1187 | -0.559 | -0.3196 | Yes |
| 19 | 5154 | PDGFA | 1191 | -0.566 | -0.2883 | Yes |
| 20 | 8408 | ULK1 | 1243 | -0.622 | -0.2884 | Yes |
| 21 | 2355 | FOSL2 | 1262 | -0.648 | -0.2631 | Yes |
| 22 | 3084 | NRG1 | 1320 | -0.739 | -0.2606 | Yes |
| 23 | 113791 | PIK3IP1 | 1345 | -0.787 | -0.2315 | Yes |
| 24 | 54790 | TET2 | 1364 | -0.842 | -0.1947 | Yes |
| 25 | 4734 | NEDD4 | 1369 | -0.856 | -0.1470 | Yes |
| 26 | 10397 | NDRG1 | 1380 | -0.925 | -0.0996 | Yes |
| 27 | 4919 | ROR1 | 1385 | -0.954 | -0.0461 | Yes |
| 28 | 26959 | HBP1 | 1395 | -1.073 | 0.0108 | Yes |
Table: GSEA details [plain text format]

  

Fig 2: FOXO1\_02      
 Blue-Pink O' Gram in the Space of the Analyzed GeneSet

  

Fig 3: FOXO1\_02: Random ES distribution      
 Gene set null distribution of ES for **FOXO1\_02**
